# Bioaccumulation of heavy metals in the different tissues of Mackerel scad, *Decapterus macarellus* (Cuvier, 1833) collected from Karachi and Gwadar Coasts of Pakistan

**DOI:** 10.1016/j.sjbs.2022.103540

**Published:** 2022-12-13

**Authors:** Saima Mehar, Iqra Anam, Zubia Masood, Sofia Alvi, Wali Khan, Muhammad Kabir, Muhammad Shahbaz, Tawseef Khan

**Affiliations:** a Department of Chemistry, Sardar Bahadur Khan Women's University (SBKWU), Quetta, Pakistan; b Department of Zoology, Sardar Bahadur Khan Women's University (SBKWU), Quetta, Pakistan; c Applied Chemistry Research Center, PCSIR Laboratory Complex, Karachi, Pakistan; d Department of Zoology, University of Malakand, Lowe Dir, Pakistan; e Department of Biological Sciences, Thal University Bhakkar, University of Sargodha (Ex-Sub Campus, Bhakkar), Bhakkar 30000, Punjab, Pakistan; f Department of Zoology, Women University of Azad Jamun and Kashmir (AJK), Bagh, Pakistan; g Department of Zoology, Abdul Wali Khan University, Mardan, KPK, Pakistan

**Keywords:** Bioaccumulation, *Decapterus macarellus*, Gwadar Coast, Heavy metals, Karachi coast, Mackerel scad

## Abstract

**Introduction:**

Among other aquatic animals, fish can also accumulate a large number of toxic metals in their various body organs, which may enter in the human body and cause serious health issues. Therefore, the basic aim of this study was to observe the level of some heavy metals (i.e., Pb, Fe, Cu, Cd, Cr) found in the different tissues of *Decapterus macarellus* collected from the Karachi and Gwadar coasts of Pakistan.

**Methods:**

About 200 fish samples of five different size groups of *Decapterus macarellus* were collected from Gwadar and Karachi fish harbors during April to September 2020. Total 10 samples of each size group i.e., S1, S2, S3, S4 & S5 were collected from each coast. Heavy metals were analyzed in fish samples by using the atomic absorption spectrophotometer (AAS).

**Results:**

The overall results revealed that some metals like Cu, Pb, Cd & Cr contents were high in the stomach, while less in the muscles. Whereas, the concentration of Fe was found to be high in the liver, while low in skin of fish. The average values of bioaccumulation of these heavy metals (BAF) were found in decreasing order of Cu > Cd > Fe > Cr > Pb. In this study, except Cd and Cr, all metals were found within the permissible limits. Both sediment and water from the selected site areas were also analyzed to observe their pollution levels in the order of; sediment > water > fish tissues.

**Conclusion:**

Thus, it was concluded that the Karachi environment was much more polluted than the Gwadar environment because it is in an industrial unit and a busy sea site for trade. Moreover, consuming muscles from this species is safe for human health except for iron toxicity, but the use of the liver is not beneficial for all selected metals. Thus, the present work will also be helpful to monitor these toxic metals in a food chain and maintain a healthy life, and reduce all kinds of health risks associated with them.

## 1 Introduction

*Decapterus macarellus* is a ray-finned fish, commonly known as mackerel scads or cigar-fish. In Pakistan, the local name of this species is “Bangra” and “Seem”. This fish belongs to the family Carangidae and sub-family Caranginae, which includes 103 species. This species is among the important seafood or oily fish, which is full of protein nutrition and prevents heart disease, diabetes, blood pressure, rheumatoid arthritis, cancer and improves immunity in humans (Titcomb 1972). This species feeds on zooplankton. *D. macarellus* is one of the unique fish as it spends its life between two marine habitats. Marine ecosystems include coral reefs and the open ocean, so it is classified as “coastal pelagic fishes”. Fisheries management has banned the hunting of this species from March to July because it is its spawning period (Khalaf et al., 2012, Flores et al., 2017). Humans use the fish as it is an important source animal proteins and minerals, which sometimes cause toxicity in them. The term heavy metal refers to the metallic element with high density with poisonous effects) (Nagajyoti et al., 2010). Heavy metals are placed among the most common and major environmental pollutants in the world (Ali et al., 2019). Nowadays, the accumulation of some toxic metals in a food chain or polluted food must be monitored to maintain a healthy life and will be helpful in reducing all kinds of health risks associated with it. Likewise, among all other animals, fish can also accumulate a large number of pollutants from their aquatic environment and other hazardous substances, and thus act as an excellent indicator of water pollution in any kind of aquatic ecosystem (Fatima et al., 2020, Sarkar and Islam, 2020). Furthermore, heavy metals are also found to have more tendency to disturb the life cycle or reproduction cycle and growth rates of fish. Moreover, these toxic metals can cause histopathological alternation of the skin or surface of gills, liver, stomach, and muscle (Abdel-Khalek et al., 2020). Heavy metals are found to decrease the plasticity effect of the cardiorespiratory response leading to in a reduced chance of fish survival under hypo-toxic conditions (Monteiro et al., 2013). Heavy metals can also accumulate in the different organs of human being such as brain, liver, kidney, lungs, and muscles when consuming these fish and produce dangerous effect on health (Isangedighi and David, 2019, Fu and Xi, 2020).

Some metallic elements like Cadmium (Cd), Chromium (Cr), Zinc (Zn), Manganese (Mn), Magnesium (Mg), Cobalt (Co), and Nickel (Ni), etc. are also called “heavy metals” because they possess a large atomic number and hence large atomic masses. Thus, heavy metals can be defined as metals much denser than water (Aycicek et al., 2008). Heavy metals are of high biological importance at trace levels (Moiseenko and Gashkina, 2020), but they can produce a toxic effect on human health if present above the permitted range, as indicated by World Health Organization (WHO) or Food and Agriculture Organization (FAO). Therefore, this issue now becomes the main focus, which prompts every scientist or nutritionist in the world to investigate the concentration of toxic levels of all aquatic organisms in an aquatic environment (Moiseenko et al., 2018). The bio-toxic effect of any heavy metals is due to its tendency to bioaccumulate in different organs of kinds of seafood or particularly fish, which in turn may enter the human body and ultimately cause serious health issues (Islam et al., 2015). These heavy metals mostly include lead, cadmium, zinc, mercury, arsenic, silver, chromium, copper, iron, and platinum group members. Few heavy metals beneficial to human health include cobalt, copper, zinc, manganese, iron, sodium, potassium, calcium, magnesium, which play a vital role in certain metabolic reactions. But, some heavy metals do not have any beneficial effect on the organisms as their presence in the living organism is considered as the main threat as they are harmful to both plants and animals (Offor et al., 2014). There are different pathways like respiration, ingestion, and absorption of water and food from their aquatic environments and their heavy metals may accumulate in the body of aquatic organisms or particularly in marine organisms (Rainbow, 2018). The bioaccumulation of heavy metals is an indicator of the increase in many human health issues (Yousafzai et al., 2012). It is a powerful tool for monitoring any kind of environmental pollution. The concentration found in fish tissue is a direct indication of water contamination with heavy metals because the organism is in direct contact with its aquatic body (Imar and Carlos, 2011). Fish bioaccumulate different pollutants. The bioaccumulation of heavy metals in fish species depends on the rate at which fish absorb heavy metals, and also depends on the metabolic activity of that species. However, fish are placed at a higher level in the food chain of any aquatic ecosystem. Thus, fish accumulate a significant concentration of heavy metals. Such an accumulation entity depends upon intake and elimination rate of fish body system (Karadede et al., 2004). Many researchers have reported that different biotic and abiotic factors are responsible for the accumulation of inorganic pollutants in fish organs and tissues. Some of these factors are type of fish species, the trophic level of fish in an ecosystem, feeding habits, age and size of fish, interspecific differences in sensitivity to various metals, concentrations of heavy metal in a water body, concentrations of heavy metal in soil sediments, the type of food on which fish feed, physical and chemical factors of water quality, nature and chemical specification of heavy metal and metal bio-availability (Griboff et al., 2018, Moiseenko and Gashkina, 2020).

The toxicity of heavy metals rises the need to investigate the content of these metals in foodstuffs such as poultry products, dairy products, and seafood, or particularly in marine fish used as food fish. Heavy metals may be part of poultry meat because the diet of poultry chicken involves the high Arsenic content in their fishmeal (Nachman et al., 2012); while in fish, heavy metals may be incorporated from their surrounding water, sediments, and diets available in their environment (Pal and Maiti, 2018, Kumar et al., 2020). According to the need of time, there is great interest in better understanding a transfer of contaminations or a transfer of heavy metals in a food chain via the food web. Thus, due to this need, it is now necessary to analyze the pollutants in a living organism over the analysis of any other abiotic factors, such as soil and water because life is more precious than any other thing (Tashla et al., 2018, Fatima et al., 2020). One of the major and main challenges for mankind and the environment is the pollution. The revolutions of the industrial sector and the increase in population are the main factors responsible for the rise in pollution levels leading to serious health issues (Da Silva & Gouveia, 2020). In Pakistan, fish is one of the favorite foods and is considered an excellent source of protein. It is liked by the majority of the nation and is believed to be associated with maintaining good health. But unfortunately, fish species are being polluted with heavy metals and these pollutants become part of the food chain and prove to be hazardous for human health (Yousif et al., 2021). The present study aims to determine the degree of pollution level and potential toxicological significance of heavy metals by analyzing fish, water, and sediment samples collected from coastal zones of Karachi and Gwadar. Therefore, in this regard, the present study was conducted on the bioaccumulation of some heavy metals in different tissues or inedible parts such as liver, stomach, flesh, and skin of mackerel scads or cigar fish *Decapterus macarellus* collected from two different sea coasts of Pakistan, which may provide significant information on the bioavailability and bio-magnification of pollutants in fish food.

## 2 Materials and methods

### 2.1 Study area

**Fig. 1:**
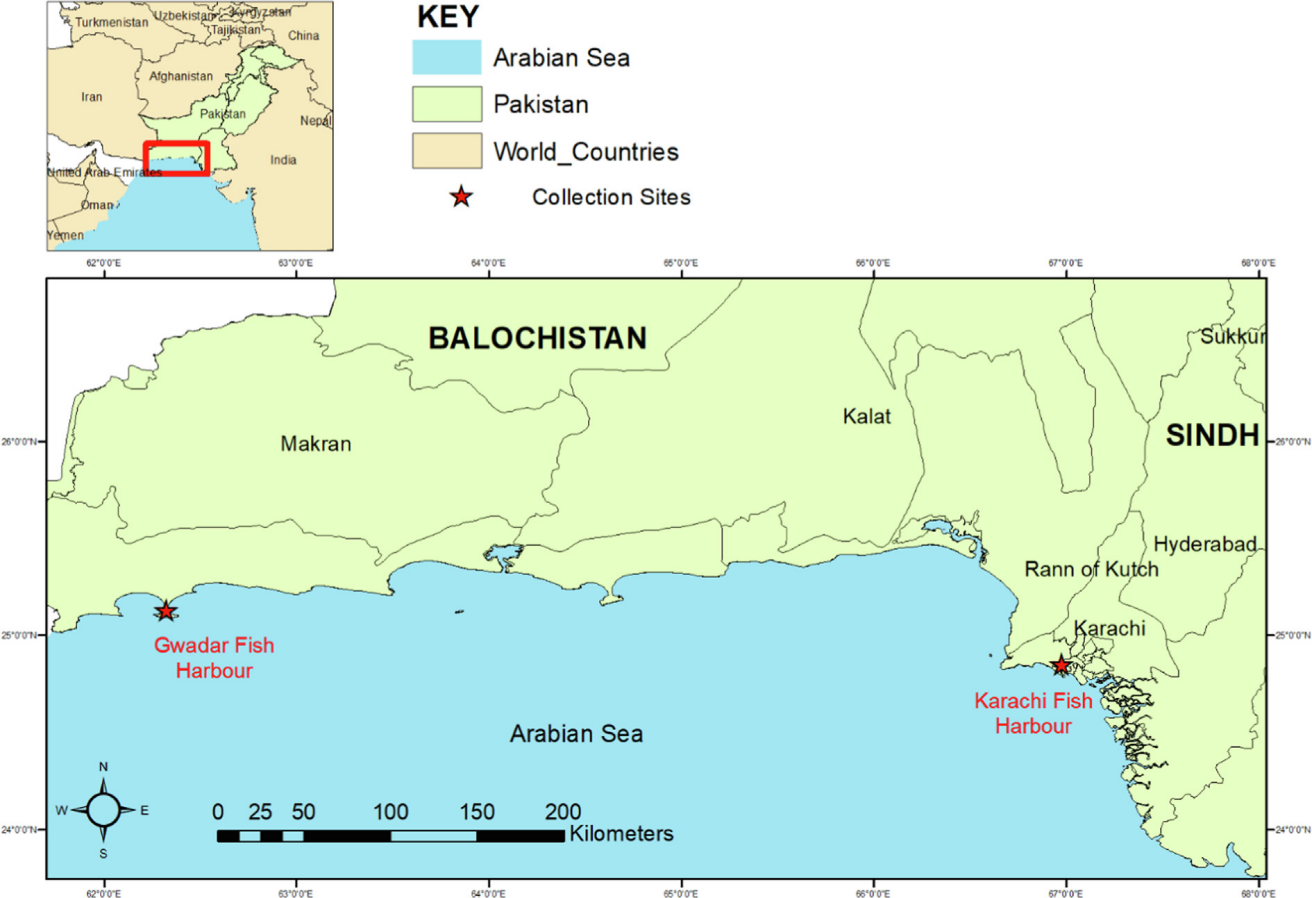
Map of Gwadar coast Pakistan.

Study areas for the present research were Karachi fish harbor (Sindh) located at 24°50′24″N, 66°58′48″E and Gwadar fish harbor (Balochistan) located at 25°7′35″N, 62°19′21″ E of Arabian sea coast of Pakistan, as shown in Fig. 1.

**Fig. 2:**
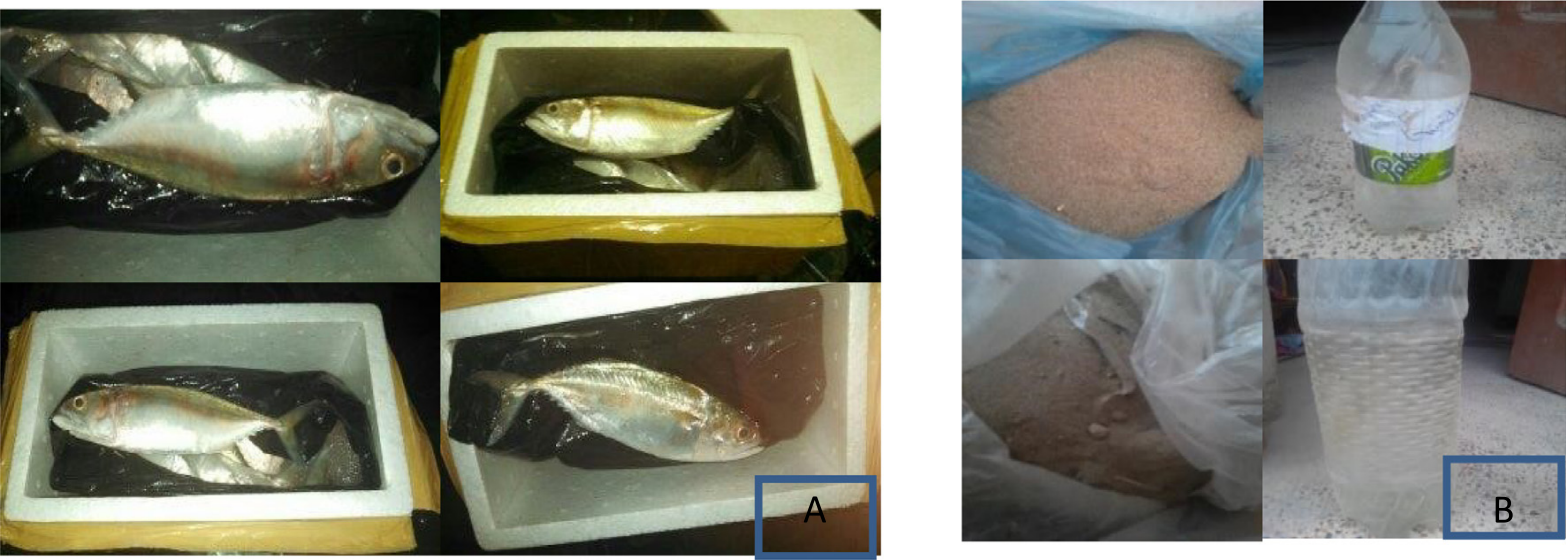
(A) Collection of fish sample. (B) Water and sediment samples.

About 200 fish samples of different size groups (S1, S2, S3, S4 & S5) of *Decapterus macarellus* with total body length (TL) ranged from 16.0 to 24.0 cm with an average value of 20.2 ± 3.19 cm were collected from landing sites at both Gwadar and Karachi fish harbors during the period from April to September 2020. The weight of fish samples collected from the Gwadar coast were found in the range between 60.4 and 300 g with an average value of 154.9 ± 94.2, while the weight of fish samples collected from the Karachi coast were ranged from 90.0 to 480.0 g with 278.0 ± 144.6 g average value, respectively. About 10 samples of each size group (i.e., S1, S2, S3, S4 & S5) were collected from each coast. Fish samples of each size group were with almost the same length were collected from each coastal site as follows; S1 = size group with average length 16 cm, S2 = size group with average length 18 cm; S3 = size group with average length 21 cm; S4 = size group with average length 22 cm; S5 = size group with average length 24 cm, respectively. All samples were collected in polythene bags as shown in Fig. 2a and were transferred to the fisheries laboratory of the Zoology department of Sardar Bahadur Khan Women's University, Quetta, Balochistan for further analysis.

### 2.2 Collection of fish organ samples

**Fig. 3:**
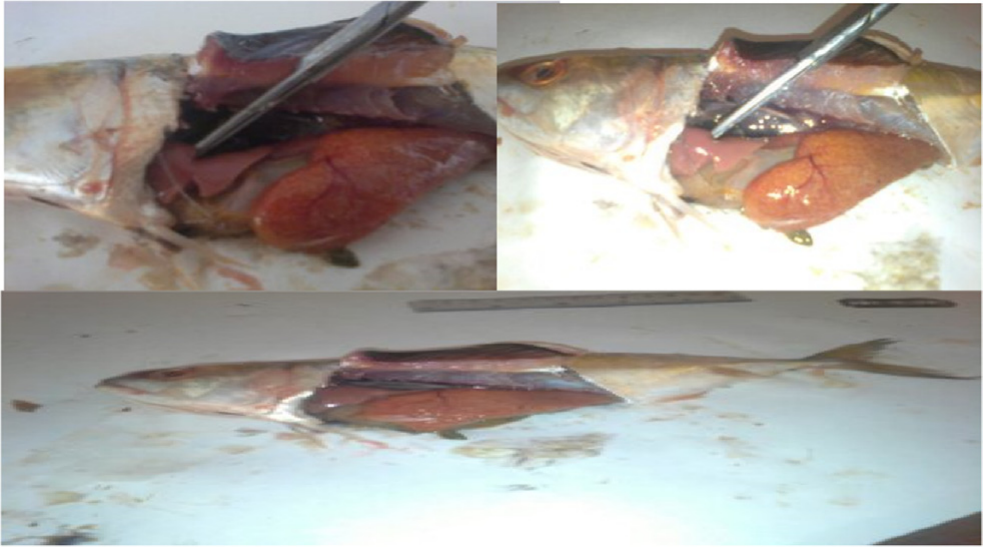
Dissection of various organs in fish samples.

Then dissection was performed with stainless steel tools as shown in Fig. 3. Distilled water was used for the washing sample. The main purpose of this experimental procedure was to separate different selected organs for analysis. These organs include the liver, flesh, skin, gills, and stomach. The first three organs are selected because it is utilized for eating purpose. Gills are selected for reason that these are vital respiratory organs in fishes, while the stomach is selected keeping in view the digestive tract that involves in the feeding process of the organism. Dissection was made from the ventral side. Gills were separated by simply removing the operculum, while other organs were collected after dissection of the abdomen. Each organ sample was weighted and then preserved in polythene beg till further analysis. For convenience, each fish sample was labeled with code such as K which stands for Karachi sample and G stands for Gwadar sample.

All organ samples separated from each fish were subjected to the oven for one hour at 110 °C till the complete dryness of the sample. A drying procedure was performed for the fact that wet samples cannot be placed for digestion because the moisture content in the organ creates difficulty in the digestive procedure. When samples were isolated through dissection, they were fresh and had enough content of water, therefore the weight of organ samples was noted after this operation. The step was necessary to obtain a sample in that form which can easily be grind and converted into powdered form.

### 2.3 Collection of water and sediment samples

Then both water and soil sediment sampling were made at both study areas. The purpose of this sampling was to determine the heavy metals contamination in water and sediment of each study area in which fish was an inhabitant. All sediment samples were collected in plastic bags and then in polythene bags. The water of each aquatic system was collected in bottles as shown in Fig. 2b). All collected samples were transferred to the Chemistry laboratory of Sardar Bahadur Khan Women's University Quetta. This analysis helps in the estimation of bioaccumulation of pollutants from the environment to the fish body by using methods as proposed by Ahmed et al., 2017, Yousif et al., 2021.

### 2.4 Analysis of moisture content of fish organ samples

Moisture content was determined by analyzing the sample weight before and after oven heating. The difference in wet weight and dry weight was a direct indication of moisture content in each organ sample by using the following equation below;

%Moisture=W2-W1/W2∗100

where W2 = Weight of sample before dry and W1 = Weight of sample after dry.

The sample was also weighted to 0.5 g after grinding for digestion. Fine powder of any analyte provides a good surface area for analysis. So, the digestion of powder-dried samples is favored for analysis. For this purpose, each dried sample was weighted to 1.5 g and then placed in mortal one by one, and grinding was achieved by using a piston. The procedure was continued till obtaining of fine powdered by following the method of Ayanda et al. (2019).

### 2.5 Digestion and determination of heavy metals contents

**Fig. 4:**
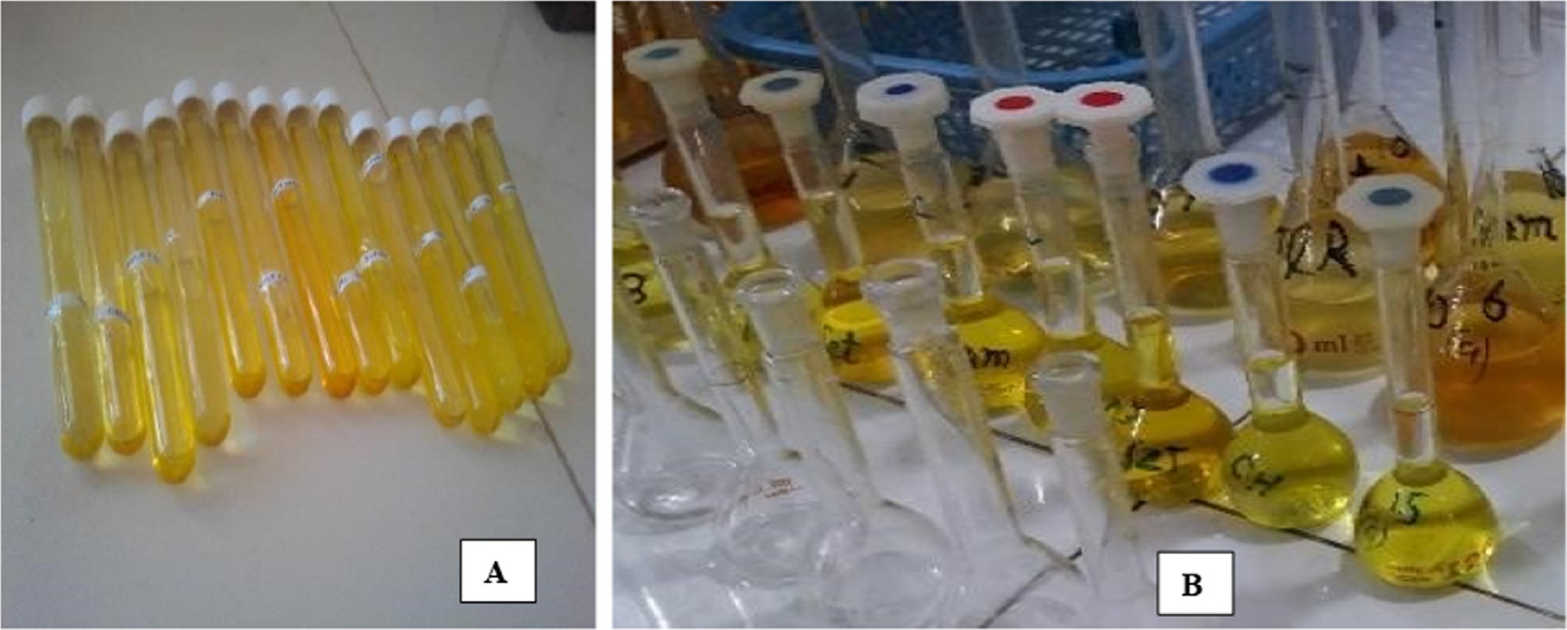
(A) Preservation of digested sample; (B) Dilution of digested samples.

Grind powdered fish tissue samples and soil sediments were placed in a conical flask and nitric acid was added to each flask till the whole sample was dipped. All samples were covered with Aluminum foil and left for two days in a clean place. Two days-soaked samples were placed on a hot plate one by one for a half-hour for complete digestion. The temperature of the hot plate was set at 60 °C. Each sample was continuously shaken for even mixing of liquid and digestion. The appearance of a clear pale-yellow mixture indicates the completion of digestion. When each sample was completely digested on a hotplate then it was placed at room temperature for 15–20 min to cool down before going for filtration. The cooled completely digested mixture of each sample was subjected for filtration through what-mann filter paper of pore size 150 mm. This step enabled me to the separation of clear digested samples from the organic residue left residue. Each sample mixture was filtered in a capped test tube so that sample can be saved till analysis. Each test tube was checked for clear appearance of sample and then caped tightly until dilution and instrumental analysis. Then 20 ml of each prepared sample was placed in a round bottle flask of 50 ml and the volume of each sample was made to 50 ml with distilled water (Fig. 4). Water samples were also digested in the same manner for the detection of these heavy metals.

The heavy metals concentration detection was analyzed using the atomic absorption spectrophotometer (AAS) supplied by polarized Zeeman atomic absorption spectrometer, model HITACHI Z-8000. Acetylene–air flame was used with methods followed by Radulescu et al. (2014) as per standard protocols given by APHA (2005). The instrument gave results in mg/L. The concentration of heavy metal in each sample was recorded with an atomic absorption spectrometer. Values were recorded in mg/L and then converter into mg/kg which is as same as ppm concentration. Following conversion formula was used for this operation. In the present research, five different organs were selected for observing the bioaccumulation of heavy metals. These selected organs were gills, skin, stomach, flesh, and liver. Samples from both field areas were analyzed and then data were comparatively analyzed to evaluate the different levels of heavy metals pollution. Results were recorded in mg/L and converted to mg/kg using this formula:

$$Heavy\;metal\;concentration\;\left(mg/kg\right)=\frac{Observed\;conc.\;\left(\frac{mg}{L}\right)\times Vol.\;of\;solution\;\left(ml\right)=ppm}{Wt.\;sample\;in\;gram}$$

All selected heavy metals were present in each analyzed sample. Analysis was made on wavelength 283.3 nm for lead, 248.3 nm for iron, 324.8 nm for copper, 540 nm for chromium, and 228.8 nm for cadmium. Data were analyzed through a standard deviation test using SPSS software version 2.0.

### 2.6 Bioaccumulation factor (BAF)

The bioaccumulation factor (BAF) of heavy metals in the five selected body tissues was also calculated in mg/L by following the equation proposed by Khalek et al. (2016) as follows;

$$BAF=\frac{Conc.\;of\;heavy\;metal\;in\;dry\;fish\;tissues\;}{Conc.\;of\;heavy\;metal\;in\;water\;samples\;of\;the\;study\;area\;}$$

### 2.7 Statistical analysis of data

The data was calculated by using MS Excel and SPSS computer software version 2.0.

## 3 Results

### 3.1 Moisture content in different tissues of fish

**Fig. 5:**
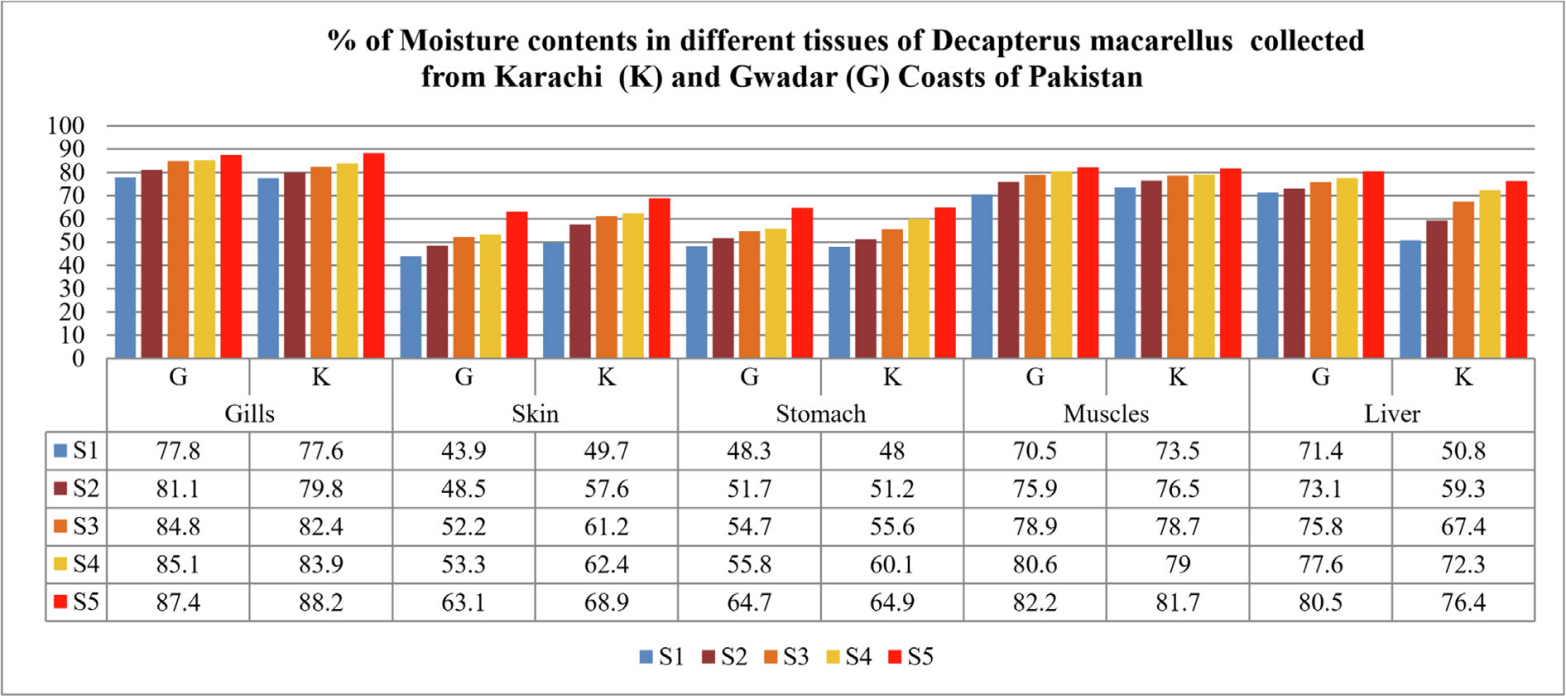
% of Moisture contents in different tissues of Decapterus macarellus collected from Karachi (k) and Gwadar (G) Coasts of Pakistan.

The percentage of moisture content calculated in the present study was found in order of; Gills > muscles > liver > skin > stomach and ranged from 43.9 to 88.2%, as shown in Table 1a, Table 1b, Table 1c, Table 1d, Table 1e and Fig. 5.

**Table 1a:** Shows the percentage of moisture content in the Gills of *Decapterus macarellus* collected from Karachi (K) and Gwadar (G) Coasts of Pakistan.

**Samples**	**Mean fish body length** **(cm) of both Gwadar and Karachi fish samples**	**Mean fish body Weight (grams)**		**Gill weight range (W2-W1) in grams**		**% Moisture in Gills of fish**	
		**G**	**K**	**G**	**K**	**G**	**K**
**S1**	16	69.4	90	6.7–1.49	6.12–1.37	77.8	77.6
**S2**	18	77.5	220	7.32–1.38	7.22–1.46	81.1	79.8
**S3**	21	142	260	7.96–1.21	10.8–1.91	84.8	82.4
**S4**	22	186	340	10.21–1.52	14.6–2.35	85.1	83.9
**S5**	24	300	480	12.8–1.61	17.4–2.05	87.4	88.2
**Mean ± S.D**	20.2 ± 3.19	157.9 ± 94.2	278 ± 144.6			83.2 ± 3.80	82.4 ± 4.07

**Table 1b:** Shows the percentage of moisture content in the Skin of *Decapterus macarellus* collected from Karachi (K) and Gwadar (G) Coasts of Pakistan.

**Samples**	**Mean fish body length** **(cm) of both Gwadar and Karachi fish samples**	**Mean fish body Weight (grams)**		**Skin weight range (W2-W1) in grams**		**% Moisture in Skin of fish**	
		**G**	**K**	**G**	**K**	**G**	**K**
**S1**	16	69.4	90	5.91–3.13	6.21–3.12	43.9	49.7
**S2**	18	77.5	220	6.82–3.51	7.11–3.01	48.5	57.6
**S3**	21	142	260	7.15–3.42	7.51–2.91	52.2	61.2
**S4**	22	186	340	7.34–3.10	8.27–3.11	53.3	62.4
**S5**	24	300	480	7.91–2.92	10.3–3.21	63.1	68.9
**Mean ± S.D**	20.2 ± 3.19	157.9 ± 94.2	278 ± 144.6			52.2 ± 7.12	59.9 ± 7.04

**Table 1c:** Shows the percentage of moisture content in the Stomach of *Decapterus macarellus* collected from Karachi (K) and Gwadar (G) Coasts of Pakistan.

**Samples**	**Mean fish body length** **(cm) of both Gwadar and Karachi fish samples**	**Mean fish body Weight (grams)**		**Stomach weight range (W2-W1) in grams**		**% Moisture in Stomach of fish**	
		**G**	**K**	**G**	**K**	**G**	**K**
**S1**	16	69.4	90	4.78–2.47	3.56–1.85	48.3	48
**S2**	18	77.5	220	5.01–2.42	4.53–2.21	51.7	51.2
**S3**	21	142	260	5.76–2.61	5.21–2.31	54.7	55.6
**S4**	22	186	340	6.82–3.01	6.29–2.51	55.8	60.1
**S5**	24	300	480	7.12–2.51	9.23–3.24	64.7	64.9
**Mean ± S.D**	20.2 ± 3.19	157.9 ± 94.2	278 ± 144.6			55.04 ± 6.14	55.9 ± 6.77

**Table 1d:** Shows the percentage of moisture content in the Muscles of *Decapterus macarellus* collected from Karachi (K) and Gwadar (G) Coasts of Pakistan.

**Samples**	**Mean fish body length** **(cm) of both Gwadar and Karachi fish samples**	**Mean fish body Weight (grams)**		**Muscles weight range (W2-W1) in grams**		**% Moisture in Muscles of fish**	
		**G**	**K**	**G**	**K**	**G**	**K**
**S1**	16	69.4	90	11.35–3.35	15.9–4.23	70.5	73.5
**S2**	18	77.5	220	21.7–5.23	26.8–6.32	75.9	76.5
**S3**	21	142	260	27.6–5.82	25.9–5.51	78.9	78.7
**S4**	22	186	340	31.4–6.10	25.8–5.41	80.6	79
**S5**	24	300	480	40.6–7.23	30.5–5.59	82.2	81.7
**Mean ± S.D**	20.2 ± 3.19	157.9 ± 94.2	278 ± 144.6			77.62 ± 4.61	77.8 ± 3.06

**Table 1e:** Shows the percentage of moisture content in the liver of *Decapterus macarellus* collected from Karachi (K) and Gwadar (G) Coasts of Pakistan.

**Samples**	**Mean fish body length** **(cm) of both Gwadar and Karachi fish samples**	**Mean fish body Weight (grams)**		**Liver weight range (W2-W1) in grams**		**% Moisture in liver of fish**	
		**G**	**K**	**G**	**K**	**G**	**K**
**S1**	16	69.4	90	7.74–2.21	3.91–1.92	71.4	50.8
**S2**	18	77.5	220	9.84–2.65	4.48–182	73.1	59.3
**S3**	21	142	260	12.91–3.12	5.12–1.67	75.8	67.4
**S4**	22	186	340	13.02–2.91	6.91–1.91	77.6	72.3
**S5**	24	300	480	15.9–3.11	9.60–2.26	80.5	76.4
**Mean ± S.D**	20.2 ± 3.19	157.9 ± 94.2	278 ± 144.6			75.68 ± 3.60	65.24 ± 10.2

### 3.2 Heavy metals concentration in different tissues of fish

In this study, Pb, Cu, Fe, Cd, and Cr content were calculated in the skin, stomach, muscles, liver, and gills of mackerel scad collected from two different selected environments, that is Karachi and Gwadar coasts. In each case, an increase in concentration with length and weight was observed. In each analysis, maximum concentration was observed in fish samples collected from the Karachi coast. During the analysis of these metal contents in gills, Fe was detected in the highest concentration among all selected metals in both aquatic environments, while Cr was found in the lowest quantity in both cases (Table 2 and Fig. 6a). The increasing order of heavy metal in gills was found in order of; Fe > Pb > Cu > Cd > Cr. Like gills, Fe was also reported in higher concentration on muscle samples, while Cd was found with minimum concentration (Table 3 and Fig. 6b). Likewise, the heavy metal profile in the skin of fish collected from Karachi and Gwadar samples in skin samples was observed in the order of; Fe > Cu > Pb > Cr > Cd (Table 4 and Fig. 6c). Unlike gills, skin, and muscle samples, Pb was found significantly high amount in stomach samples of fishes collected from both coasts (Table 5 and Fig. 6d). The increasing order of selected metals in fish stomach samples for Karachi coast was Pb > Fe > Cu > Cr > Cd, but for Gwadar coast was Pb > Fe > Cu > Cd > Cr. Comparatively, the Karachi sample contains a high concentration of metals as compared to the Gwadar sample which indicates more metal pollution in the Karachi aquatic body as compared to the Gwadar aquatic ecosystem (Fig. 6a, Fig. 6b, Fig. 6c, Fig. 6d, Fig. 6e). As the liver is among the vital organs of living organisms, therefore, in the present study, iron was observed with the highest accumulation in liver samples of fishes collected from both Karachi and Gwadar coasts. The increasing order of selected metals in the liver from both aquatic bodies was observed as, Fe > Pb > Cu > Cd > Cr as mentioned in Table 6 and Fig. 6e. Bioaccumulation of selected metals found in different organs was summarized in Table 7. Table 8 shows the average range of metals in the different tissues of *Decapterus macarellus* collected from Gwadar and Karachi Coast with permissible limits of these metals according to FAO/WHO guidelines of fish quality. The overall results revealed that Le in the stomach (K), Cd in gills, liver, stomach (K & G), and skin (K), while Cr was also found in all tissues beyond the permissible range of FAO/WHO limits. However, both Fe and Cu were found in the permissible range. The overall results revealed that most heavy metals like Cu, Pb, Cd & Cr contents were high in the stomach, while less in muscles. Whereas, the concentration of iron Fe was found to be high in the liver, while low in the skin of fishes. Table 9 shows the bioaccumulation of heavy metals in each body tissue. The overall results show the accumulation of heavy metals (BAF) average values were found in decreasing order of, Cu > Cd > Fe > Cr > Pb, respectively. Such observation showed that muscles, which are the most favorite part of food are least susceptible to metal accumulation.

**Table 2:** Shows the concentration of metals in Gills of *Decapterus macarellus* collected from Karachi and Gwadar Coasts of Pakistan.

	**Fish body length (TL)**	**Lead**		**Copper**		**Iron**		**Cadmium**		**Chromium**	
**Fish body length (TL)**		**mg/kg**		**mg/kg**		**mg/kg**		**mg/kg**		**mg/kg**	
**Size group**		**K**	**G**	**K**	**G**	**K**	**G**	**K**	**G**	**K**	**G**
	**Mean length (cm)**	**Mean ± S.D**	**Mean ± S.D**	**Mean ± S.D**	**Mean ± S.D**	**Mean ± S.D**	**Mean ± S.D**	**Mean ± S. D**	**Mean ± S.D**	**Mean ± S. D**	**Mean ± S.D**
S1	16	0.9 ± 0.05	0.4 ± 0.01	0.9 ± 0.005	0.7 ± 0.01	2.3 ± 0.10	1.9 ± 0.03	0.5 ± 0.01	0.3 ± 0.03	0.7 ± 0.02	0.5 ± 0.15
S2	18	1.1 ± 0.11	1.0 ± 0.05	1.1 ± 0.11	1.1 ± 0.028	3.6 ± 0.07	2.9 ± 0.01	0.9 ± 0.01	0.7 ± 0.1	0.9 ± 0.15	1.1 ± 0.15
S3	21	2.1 ± 0.05	1.1 ± 0.01	2.1 ± 0.52	2.0 ± 0.05	3.9 ± 0.05	3.3 ± 0.04	1.6 ± 0.05	1.9 ± 0.05	1.0 ± 0.17	1.2 ± 0.04
S4	22	2.5 ± 0.11	2.1 ± 0.05	2.5 ± 0.05	2.3 ± 0.05	6.2 ± 0.06	5.8 ± 0.06	2.8 ± 0.04	2.6 ± 0.005	1.5 ± 0.03	2.5 ± 0.02
S5	24	3.2 ± 0.05	2.9 ± 0.01	3.1 ± 0.02	2.7 ± 0.02	7.9 ± 0.015	6.2 ± 0.03	3.9 ± 0.01	3.1 ± 0.01	2.0 ± 0.05	2.8 ± 0.02
**Mean ± S.D**	20.2 ± 3.19	1.96 ± 0.9	1.5 ± 0.9	1.94 ± 0.93	1.76 ± 0.84	4.78 ± 2.24	4.02 ± 1.88	1.94 ± 1.40	1.72 ± 1.20	1.22 ± 0.53	1.62 ± 0.98

Note: *S.D = standard deviation; K = Karachi coast; G = Gwadar Coast.

**Fig. 6a:**
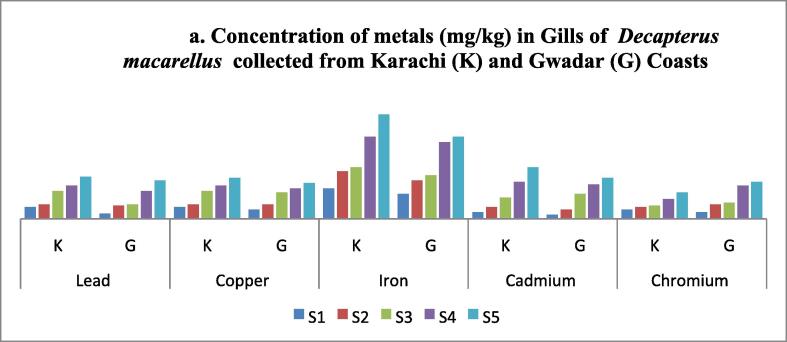
Concentration of metals (mg/kg) in Gills of *Decapterus macarellus* collected from Karachi (K) and Gwadar (G) Coasts.

**Table 3:** Shows the concentration of metals in Muscles of *Decapterus macarellus* collected from Karachi and Gwadar Coasts of Pakistan.

	**Fish body** **length (TL)**	**Lead**		**Copper**		**Iron**		**Cadmium**		**Chromium**	
**Fish body** **length (TL)**	**Average length in cm**	**mg/kg**		**mg/kg**		**mg/kg**		**mg/kg**		**mg/kg**	
**Size group**		**K**	**G**	**K**	**G**	**K**	**G**	**K**	**G**	**K**	**G**
S1	16	0.8 ± 0.01	0.3 ± 0.01	0.8 ± 0.05	0.3 ± 0.09	2.7 ± 0.10	3.1 ± 0.005	0.1 ± 0.01	0.1 ± 0.05	0.2 ± 0.02	0.2 ± 0.03
S2	18	1.2 ± 0.02	0.4 ± 0.02	1.8 ± 0.04	0.6 ± 0.09	4.0 ± 0.17	3.9 ± 0.08	0.2 ± 0.03	0.1 ± 0.02	0.2 ± 0.07	0.4 ± 0.06
S3	21	1.5 ± 0.01	0.8 ± 0.01	2.0 ± 0.11	1.1 ± 0.03	4.8 ± 0.05	4.4 ± 0.05	0.3 ± 0.01	0.2 ± 0.01	0.6 ± 0.02	0.4 ± 0.05
S4	22	1.6 ± 0.02	0.9 ± 0.01	1.9 ± 0.01	1.3 ± 0.05	6.1 ± 0.05	4.7 ± 0.01	0.4 ± 0.09	0.2 ± 0.02	1.2 ± 0.01	0.5 ± 0.05
S5	24	2.1 ± 0.04	1.4 ± 0.01	2.4 ± 0.05	1.8 ± 0.04	6.5 ± 0.02	5.2 ± 0.03	0.4 ± 0.05	0.3 ± 0.03	1.7 ± 0.04	0.8 ± 0.07
**Mean ± S.D**	20.2 ± 3.19	1.44 ± 0.48	0.76 ± 0.44	1.78 ± 0.59	1.02 ± 0.59	4.28 ± 1.55	4.26 ± 0.80	0.28 ± 0.13	0.18 ± 0.08	0.78 ± 0.66	0.46 ± 0.22

Note: *S.D = standard deviation; K = Karachi coast; G = Gwadar Coast.

**Fig. 6b:**
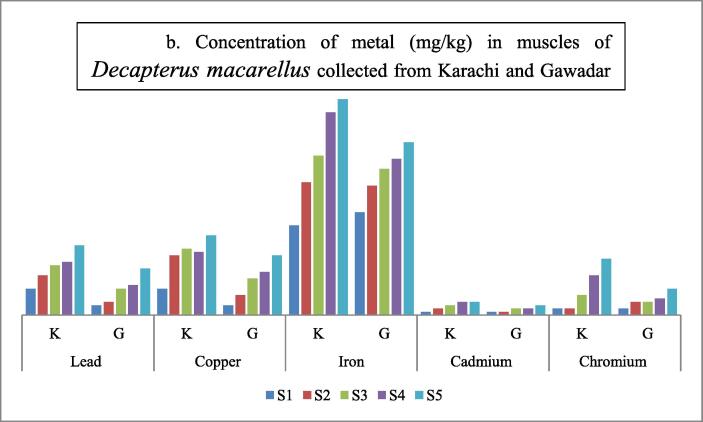
Concentration of metal (mg/kg) in muscles of Decapterus macarellus collected from Karachi and Gawadar.

**Table 4:** Shows the concentration of metals in skin of *Decapterus macarellus* collected from Karachi and Gwadar Coasts of Pakistan.

	**Fish body length (TL)**	**Lead**		**Copper**		**Iron**		**Cadmium**		**Chromium**	
**Fish body length (TL)**	**Average length in cm**	**mg/kg**		**mg/kg**		**mg/kg**		**mg/kg**		**mg/kg**	
**Size group**		**K**	**G**	**K**	**G**	**K**	**G**	**K**	**G**	**K**	**G**
**S1**	16	1.2 ± 0.05	0.5 ± 0.02	2.0 ± 0.05	1.1 ± 0.02	2.4 ± 0.01	1.6 ± 0.01	0.7 ± 0.01	0.2 ± 0.02	0.9 ± 0.07	0.7 ± 0.09
**S2**	18	1.4 ± 0.04	0.9 ± 0.005	2.3 ± 0.02	1.9 ± 0.04	2.9 ± 0.02	2.4 ± 0.03	0.9 ± 0.07	0.3 ± 0.02	1.1 ± 0.055	0.8 ± 0.06
**S3**	21	2.0 ± 0.01	1.2 ± 0.03	3.1 ± 0.04	2.6 ± 0.02	3.6 ± 0.01	2.8 ± 0.03	1.2 ± 0.1	0.5 ± 0.04	1.6 ± 0.05	1.2 ± 0.04
**S4**	22	2.1 ± 0.02	1.6 ± 0.03	3.5 ± 0.02	3.1 ± 0.03	3.9 ± 0.02	3.1 ± 0.03	1.3 ± 0.04	0.6 ± 0.51	1.8 ± 0.02	1.4 ± 0.01
**S5**	24	2.5 ± 0.02	1.9 ± 0.03	4.1 ± 0.07	3.6 ± 0.02	4.1 ± 0.11	3.6 ± 0.005	1.6 ± 0.05	0.9 ± 0.02	2.1 ± 0.026	1.7 ± 0.05
**Mean ± S.D**	20.2 ± 3.19	1.84 ± 0.53	1.22 ± 0.55	3.0 ± 0.86	2.46 ± 0.99	3.38 ± 0.71	2.70 ± 0.75	1.14 ± 0.35	0.5 ± 0.27	1.5 ± 0.49	1.16 ± 0.42

Note: *S.D = standard deviation; K = Karachi coast; G = Gwadar Coast.

**Fig. 6c:**
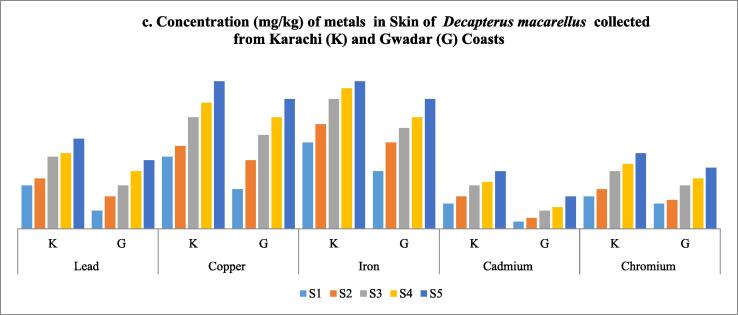
Concentration (mg/kg) of metals in Skin of Decapterus macarellus collected from Karachi (K) and Gwadar (G) Coasts.

**Table 5:** Shows the concentration of metals in stomach of *Decapterus macarellus* collected from Karachi and Gwadar Coasts of Pakistan.

		**Lead**		**Copper**		**Iron**		**Cadmium**		**Chromium**	
**Fish body length (TL)**	**Average Body length in cm**	**mg/kg**		**mg/kg**		**mg/kg**		**mg/kg**		**mg/kg**	
**Size group**		**K**	**G**	**K**	**G**	**K**	**G**	**K**	**G**	**K**	**G**
S1	16	3.6 ± 0.03	1.9 ± 0.01	2.1 ± 0.01	0.8 ± 1.31	2.8 ± 0.08	0.6 ± 0.07	2.6 ± 0.11	0.6 ± 0.02	0.9 ± 0.02	0.4 ± 0.01
S2	18	4.3 ± 0.01	2.6 ± 0.01	2.9 ± 0.05	1.3 ± 0.01	3.8 ± 0.02	1.8 ± 0.015	3.1 ± 0.057	0.8 ± 0.02	1.2 ± 0.02	0.8 ± 0.01
S3	21	8.7 ± 0.03	3.2 ± 0.01	3.5 ± 0.01	1.9 ± 0.09	5.3 ± 0.03	2.9 ± 0.01	3.5 ± 0.08	1.5 ± 0.05	1.8 ± 0.03	1.4 ± 0.02
S4	22	9.2 ± 0.02	3.7 ± 0.03	4.1 ± 0.05	2.0 ± 0.01	5.8 ± 0.01	3.3 ± 0.05	3.7 ± 0.05	1.9 ± 0.04	2.4 ± 0.025	1.8 ± 0.02
S5	24	11.2 ± 0.01	4.5 ± 0.04	5.9 ± 0.01	2.4 ± 0.04	6.3 ± 0.03	4.1 ± 0.05	4.1 ± 0.1	2.5 ± 0.03	3.1 ± 0.015	2.1 ± 0.01
**Mean ± S.D**	20.2 ± 3.19	7.04 ± 3.29	3.18 ± 1.0	3.7 ± 1.4	1.68 ± 0.63	4.8 ± 1.46	2.54 ± 1.36	3.40 ± 0.57	1.46 ± 0.78	1.88 ± 0.89	1.3 ± 0.7

Note: *S.D = standard deviation; K = Karachi coast; G = Gwadar Coast.

**Fig. 6d:**
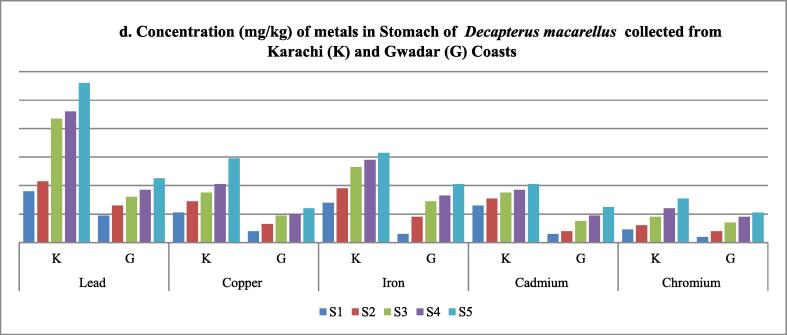
Concentration (mg/kg) of metals in Stomach of Decapterus macarellus collected from Karachi (K) and Gwadar (G) Coasts.

**Fig. 6e:**
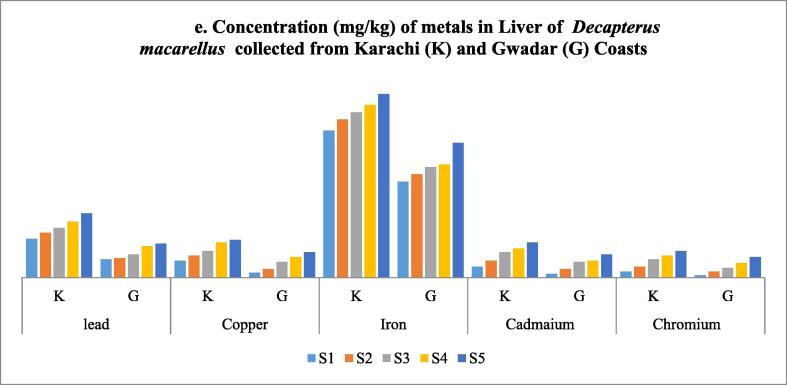
Concentration (mg/kg) of metals in Liver of Decapterus macarellus collected from Karachi (K) and Gwadar (G) Coasts.

**Table 6:** Shows the concentration of metals in the liver of *Decapterus macarellus* collected from Karachi and Gwadar Coasts of Pakistan.

	**Fish body length (TL)**	**Lead**		**Copper**		**Iron**		**Cadmium**		**Chromium**	
**Fish body length (TL)**	**Average length in cm**	**mg/kg**		**mg/kg**		**mg/kg**		**mg/kg**		**mg/kg**	
**Size group**		**K**	**G**	**K**	**G**	**K**	**G**	**K**	**G**	**K**	**G**
S1	16	3.6 ± 0.02	1.9 ± 0.01	1.4 ± 0.09	0.4 ± 0.05	12.1 ± 0.5	7.9 ± 0.3	0.9 ± 0.02	0.3 ± 0.02	0.5 ± 0.54	0.2 ± 0.05
S2	18	4.3 ± 0.01	2.6 ± 0.005	1.8 ± 0.005	0.7 ± 0.01	13.0 ± 0.01	8.5 ± 0.1	1.4 ± 0.05	0.7 ± 0.05	0.9 ± 0.02	0.5 ± 0.01
S3	21	5.3 ± 0.32	3.2 ± 0.01	2.2 ± 0.02	1.3 ± 0.04	13.6 ± 0.2	9.1 ± 0.3	2.1 ± 0.1	1.3 ± 0.03	1.5 ± 0.02	0.8 ± 0.1
S4	22	6.2 ± 0.20	3.7 ± 0.03	2.9 ± 0.02	1.7 ± 0.04	14.2 ± 0.5	9.3 ± 0.2	2.4 ± 0.02	1.4 ± 0.05	1.8 ± 0.01	1.2 ± 0.05
S5	24	7.8 ± 0.1	4.5 ± 0.04	3.1 ± 0.1	2.1 ± 0.07	15.1 ± 1.0	11.1 ± 0.1	2.9 ± 0.02	1.9 ± 003	2.2 ± 0.06	1.7 ± 0.01
**Mean ± S.D**	20.2 ± 3.19	4.18 ± 0.18	2.08 ± 0.59	2.28 ± 0.72	1.24 ± 0.7	13.6 ± 1.14	9.18 ± 1.20	1.94 ± 0.8	1.12 ± 0.63	1.38 ± 0.68	0.88 ± 0.59

Note: *S.D = standard deviation; K = Karachi coast; G = Gwadar Coast.

**Table 7:** Ranking of fish body organs of *Decapterus macarellus* according to their significant potential in bio-accumulation of heavy metals.

**Rank**	**Cu**		**Pb**		**Fe**		**Cd**		**Cr**	
	K	G	**K**	**G**	**K**	**G**	**K**	**G**	**K**	**G**
**1**	Stomach	Stomach	Stomach	Stomach	Liver	Liver	Stomach	Stomach	Stomach	Gills
**2**	Skin	Skin	Liver	Liver	Stomach	Stomach	Liver	Liver	Liver	Stomach
**3**	Liver	Liver	Gills	Gills	Gills	Gills	Gills	Gills	Skin	Skin
**4**	Gills	Gills	Skin	Skin	muscles	muscles	Skin	Skin	Gills	Liver
**5**	muscles	muscles	muscles	muscles	Skin	Skin	muscles	muscles	muscles	muscles

**Table 8:** Shows average metal concentration in different tissues of *Decapterus macarellus* collected from Gwadar and Karachi Coast with fish quality guidelines of standards values according to WHO/FAO.

**Metal**	**Average Observed values in Gills (mg/kg)**		**Average Observed values in Muscles (mg/kg)**		**Average Observed values in Skin (mg/kg)**		**Average Observed values in Stomach (mg/kg)**		**Average Observed values in Liver (mg/kg)**		**Standard values/WHO/FAO**	**References**
	**K**	**G**	**K**	**G**	**K**	**G**	**K**	**G**	**K**	**G**	mg/kg	
**Pb**	1.96 ± 0.9	1.5 ± 0.9	1.44 ± 0.48	0.76 ± 0.44	1.84 ± 0.53	1.22 ± 0.55	7.04 ± 3.29*	3.18 ± 1.0	4.18 ± 0.18	2.08 ± 0.59	5.0	FAO, 1989, Kaur et al., 2018
**Cu**	1.94 ± 0.93	1.76 ± 0.84	1.78 ± 0.59	1.02 ± 0.59	3.0 ± 0.86	2.46 ± 0.99	3.7 ± 1.4	1.68 ± 0.63	2.28 ± 0.72	1.24 ± 0.7	10.0	Kaur et al., 2018
**Fe**	4.78 ± 2.24	4.02 ± 1.88	4.28 ± 1.55	4.26 ± 0.80	3.38 ± 0.71	2.70 ± 0.75	4.8 ± 1.46	2.54 ± 1.36	13.6 ± 1.14	9.18 ± 1.20	100	FAO/WHO, 1989
**Cd**	1.94 ± 1.40*	1.72 ± 1.20*	0.28 ± 0.13	0.18 ± 0.08	1.14 ± 0.35*	0.5 ± 0.27	3.40 ± 0.57*	1.46 ± 0.78*	1.94 ± 0.80*	1.12 ± 0.63*	1.0	Kaur et al., 2018
**Cr**	1.22 ± 0.53*	1.62 ± 0.98*	0.78 ± 0.66*	0.46 ± 0.22*	1.5 ± 0.49*	1.16 ± 0.42*	1.88 ± 0.89*	1.3 ± 0.70*	1.38 ± 0.68*	0.88 ± 0.59*	0.05	Kaur et al., 2018

Note: shows the Mean value of a metal in the column with * reveal the concentrations of heavy metals above the permissible limits.

**Table 9:** Bioaccumulation Factor (BAF) for analyzed metals calculated in mg/kg for the different tissues of *Decapterus macarellus* collected from Karachi and Gwadar Coasts of Pakistan.

**Metals**	**BAF (Gills)**		**BAF (Muscles)**		**BAF (Skin)**		**BAF (Stomach)**		**BAF (Liver)**		**Mean ± S.D**
	K	G	K	G	K	G	K	G	K	G	
**Pb**	0.09^e^ (0.9–3.2)	0.09^e^ (0.4–2.9)	0.07^f^ (0.8–2.1)	0.04^g^ (0.3–1.4)	0.09^e^ (1.2–2.5)	0.07^f^ (0.5–1.9)	0.34^a^ (3.6–11.2)	0.18^c^ (1.9–4.5)	0.19^b^ (3.2–5.3)	0.12^d^ (1.5–2.8)	0.13 ± 0.09*
**Cu**	0.35^g^ (0.9–3.1)	0.46^d^ (0.7–2.7)	0.32^i^ (0.8–2.4)	0.27^j^ (0.3–1.8)	0.53^c^ (2.0–4.1)	0.65^a^ (1.1–3.6)	0.66^b^ (2.1–5.9)	0.44^e^ (0.8–2.4)	0.41^f^ (1.4–3.1)	0.33^h^ (0.4–2.1)	0.44 ± 0.14**
**Fe**	0.13^d^ (2.3–7.9)	0.14^c^ (1.9–6.2)	0.13^d^ (2.7–6.5)	0.14^c^ (3.1–5.2)	0.08e (2.4–4.1)	0.09^e^ (1.6–3.6)	0.13^d^ (2.8–6.3)	0.09^e^ (0.6–4.1)	0.36^a^ (12.1–15.1)	0.31^b^ (7.9–11.1)	0.16 ± 0.09
**Cd**	0.30^b^ (0.5–3.9)	0.29^c^ (0.3–3.1)	0.04^h^ (0.1–0.4)	0.03^i^ (0.1–0.3)	0.18^f^ (0.7–1.6)	0.08^g^ (0.2–0.9)	0.53^a^ (2.6–4.1)	0.25^d^ (0.6–2.5)	0.30^b^ (0.9–2.9)	0.19^e^ (0.3–1.9)	0.29 ± 0.15
**Cr**	0.15^e^ (0.7–2.0)	0.25^a^ (0.5–2.8)	0.09^g^ (0.2–1.7)	0.07^h^ (0.2–0.8)	0.19^c^ (0.9–2.1)	0.18^d^ (0.7–1.7)	0.23^b^ (0.9–3.1)	0.19^c^ (0.4–2.1)	0.17e (0.5–2.2)	0.13^f^ (0.2–1.7)	0.16 ± 0.06

**Note:** All values of heavy metals concentration are in mg/L; N value represents correlation (R^2^) is negative. Superscripts indicating the accumulation of heavy metals in different tissues of Makerel scad. Bracket shows the range of heavy metal found in a tissue. ** shows high mean value, * lowest mean value of bioaccumulation of a metal in different tissues.

### 3.3 Heavy metals concentration in water and sediments

**Fig. 7:**
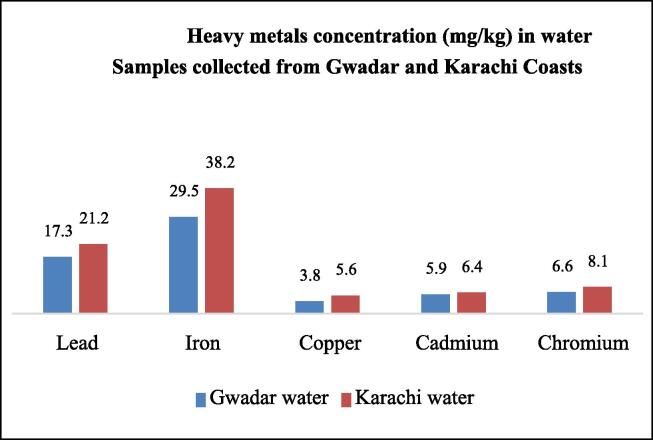
Heavy metals concentration (mg/kg) in water Samples collected from Gwadar and Karachi Coasts.

**Fig. 8:**
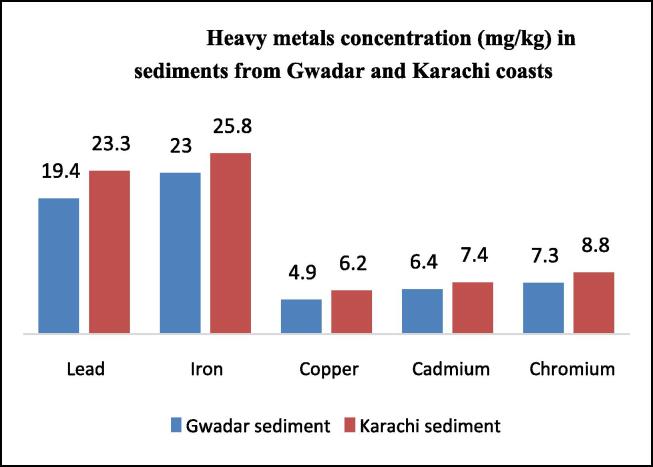
Heavy metals concentration (mg/kg) in sediments from Gwadar and Karachi coasts.

The concentration of heavy metals in water and sediment samples for both Karachi and Gwadar coasts was also analyzed and their results were presented in Table 10, Figs. 7, and 8. All metals were found beyond the permissible limits. A higher concentration of each metal was present in the water of the Karachi site as compared to Gwadar. It is probably due to fact that Karachi is an industrial center while Gwadar is not. Most pollutants are discharged into the aquatic system through industrial waste. The maximum concentration was found to be Fe. The metal which was noticed in the lowest concentration was Cd. The observed increasing order was Fe > Pb > Cu > Cr > Cd. The concentration of each heavy metal in all water samples exceeds the permissible limit as set by WHO. Thus, present research revealed that the use of water in both ecosystems is not good for fish health. The sediment acts as a sink for every pollutant, which encounters any aquatic body. Thus, sediments of both study areas were analyzed for the determination of heavy metals. The concentration of all selected metals in sediments was the same as found in water samples in order of; Fe > Pb > Cu > Cr > Cd, but the concentration of heavy metal in water samples was less than the concentration found in sediment analysis. It proves that sediment act as a sink for the aquatic system. Even maximum concentration was noticed in sediment samples as compared to water and fish organs samples.

**Table 10:** Concentration of heavy metals in water and sediment samples collected from Gwadar and Karachi Coasts of Pakistan.

**Metal**	**Water samples (mg/kg)**		**Sediments (mg/kg)**		**WHO limit (mg/kg or ppm)**
	**G**	**K**	**G**	**K**	
	**Mean ± S.D**	**Mean ± S.D**	**Mean ± S.D**	**Mean ± S.D**	
**Pb**	17.32 ± 0.03	21.21 ± 0.01	19.42 ± 0.05	23.32 ± 0.02	0.50
**Cu**	6.62 ± 0.005	8.12 ± 0.05	7.28 ± 0.01	8.77 ± 0.03	2.0
**Fe**	22.52 ± 0.01	24.21 ± 0.06	23.01 ± 0.01	25.76 ± 0.07	0.3
**Cd**	3.81 ± 0.05	5.64 ± 0.04	4.95 ± 0.02	6.15 ± 0.05	0.7
**Cr**	5.93 ± 0.02	6.83 ± 0.03	6.41 ± 0.04	7.39 ± 0.04	0.1

## 4 Discussion

Fish is an excellent source of highly digestible proteins, vitamins, minerals, and poly-unsaturated fatty acids. The biochemical composition of fish includes 66–81% water, 16–21% protein, <0.5% carbohydrates, 0.2–25% lipids, and 1.2–1.5% minerals. However, such a biochemical composition could vary between different populations of the same or different species due to seasonal changes and environmental conditions of their habitat or variations in their feeding habits, reproduction, sexual maturity, etc. (Yeannes and Almandos, 2003). In the present study, the percentage of moisture content in different body tissues ranged from 43.9 to 88.2%, as shown in Fig. 5, respectively. This result revealed that the moisture content is the major component of the edible part of fish and could play a vital role in certain metabolic reactions, and also may help solubilize various elements easily. Moreover, such high moisture contents may influence the degradation of certain polyunsaturated fatty acids, and increase the vulnerability of fish to decomposition by certain microorganisms, therefore reducing the fish quality for its prolonged storage, as reported by Abass et al. (2012).

Fish can easily bio-accumulate various kinds of heavy metals in their bodies from water, sediments, and aquatic foods in their environment. Although some of these heavy metals are valuable for fish growth as well as contribute to its metabolic reactions, however, overthrown all these health benefits on their toxicity which can also further affect the human population in its food chain from the kidney to liver damages, cardiovascular diseases and in certain extreme cases may lead to death, as previously reported by Al-Busaidi et al. (2011), and Rahman et al. (2012). As the rising demand for fish meat is not only due to its taste, but also because of its nutritional values, easily digestible protein, source of essential amino acids like cysteine, polyunsaturated fatty acids present in adequate quantities in their body tissues that have the ability to both prevent and cure various human diseases including cancers, rheumatoid arthritis, heart diseases, and inflammation. Moreover, minerals found in fish are also involved in catalyzing various metabolic reactions, controlling osmotic balance, and helping in forming bones (Raatz et al., 2013, Ayanda et al., 2019). Heavy metals found in any aquatic body may produce terrible effects on the normal range of certain environmental factors as well as the diversity of aquatic species.

Vosylienė and Jankaitė (2006) reported that sewage discharge, emission from automobiles, oil refineries, waste discharges from industries, certain metallurgical activities, and agricultural production are the main sources of anthropogenic heavy metal pollution of any aquatic environment. When all these heavy metals bio accumulate in the aquatic food chain than harm organisms and cause death in extreme cases. It is therefore essential to examine the impact of these heavy metals on the various organs of the fish body. Therefore, the nutritional composition of fish is of extreme significance to ensure that whether fish is meet up to all standard levels with the requirements of food regulations and commercial recommendations as described by FAO (1983). Olusola and Aiyesanmi (2005) reported that when fish are exposed to heavy metals, their organs mostly bioaccumulate all of these metals at varying concentrations. Ayanda et al. (2019) had detected that no single fish species can accumulate all kinds of selected metals at a similar concentration during the study, for example, they observed Ni and Zi were detected in *Oreochromis niloticus*, but detected in remaining species like *Parachanna obscura, Malapterurus electricus*, and *Chrysichthys nigrodigitatus.* However, Le was detected in *Oreochromis niloticus*, but not in the remaining three fish species. Moreover, Ma was only found in *Chrysichthys nigrodigitatus*. Such variation in the concentration of metals might be due to their different metabolic rates, the amount of food consumed by these species as well as their food requirements, as reported by Ademoroti (1996). Any of these factors can be played a vital role in creating the differences observed in the bioaccumulation of metals among different fish species. Likewise, Adewumi et al. (2014) had also reported the activities of microorganisms in the water, as well as the immediate interaction with aquatic habitat, feeding rate, feeding habits, and age of fish, are also some other factors that can influence metal concentrations between fish organs or within fish species in their aquatic habitats. Bioaccumulation of heavy metals is one of the monitoring factors use to monitor the health of the aquatic environment (Carla et al., 2004) as there is a direct attraction of aquatic organisms with their environment. Thus, living aquatic organisms are in direct contact with contaminated water. The concentration of heavy metals in the tissues of fish species is a good indicator to evaluate metal contamination (Al-Kahtani, 2009). Heavy metals accumulate in fish via water, sediments, food such as algae upon which both herbivorous and omnivorous fish feed on. In the present research, heavy metal pollution in fish species, *Decapterus macarellus* is observed to assess the pollution level in two aquatic ecosystems of Pakistan, Karachi and Gwadar coasts. Both are economically important and our research is an indirect estimation of pollution in these water bodies through analyzing fish which act as bio-indicators. The purpose of this research is based on the high consumption of fish as foodstuff by Asian people. If contaminated food is going to be consumed so, it will impart a negative health impact. All metals were present in higher concentrations on Karachi samples, which is probably due to industrial unit and trading water body. Ayas et al. (2007) had also reported a higher concentration of heavy metals in sediment samples as compared to water and fish body organs. This study also aimed to raise awareness of the health risks associated with the use of contaminating foods from the adjoining area of these sites located in Pakistan. The study also highlighted the source of pollution in this aquatic body, which may be helpful in suggesting some possible ways to reduce pollution to a low level. Anyhow, the selected fish was not having any metal above the permissive limit except for Fe. The research also showed a higher concentration in the liver, not in flesh, so it is safe to eat this fish (*Decapterus macarellus*). Karachi is one of the largest business hubs of Pakistan, situated along the Arabic sea and in the southern part of Pakistan. As it is a business hub, it is one of the most polluted cities that has a 167 km long coastal area. Many problems arose due to the rapid industrialization in the city. Urbanization and industrialization have affected the environmental quality and disturbed the natural ecological system. A reported estimation is that more than 6000 industries of Karachi may cause a release of untreated discharge of more than 300 million gallons per day (MGD) into both Layari and Malir Rivers, which ultimately transfer this polluted waste into the Arabian sea (Siddique et al. 2009). Gwadar is one of the main and significant ports of Pakistan and the center of attraction due to CEPEC. It is historically used for trade between different parts of the world. It is part of the southwestern coastal side of Balochistan and is placed among a port city of Pakistan. Gwadar is about 700 Km from Karachi and is located east of the Persian Gulf and opposite Oman. The local population of Gwadar mostly consumed fish as food as they were easily available here. However, the fuel used in different shipping boats is one of the main reasons to expose Gwadar aquatic body to heavy metal pollution and ambulating in fish species found in it.

## 5 Conclusion

Data obtained from this research showed that fish muscles were the organ in which a minimum concentration of heavy metals was observed. This is of great importance because muscles contributes the greatest mass of flesh consumed as food for man and other animals. Results also revealed that Karachi's environment was much more polluted than Gwadar's environment as it is an industrial unit and a busy maritime site with special attention for trade. These two factors are responsible for the discharge of heavy metals into an aquatic ecosystem. The results also showed a higher concentration of heavy metals in sediments samples. The order observed for heavy metals bioaccumulation in each aquatic ecosystem was as follows: sediments > water > fish organs. Heavy metal bioaccumulated concentration is also directly proportional to the increasing length of fish.

## Funding

Not Applicable. This research work was not funded by any funding agency or other resources.

## Ethics approval and consent to participate

The study protocol and the ethics of this work have been approved by the Ethical Committee of the Sardar Bahadur Khan Women's University Quetta.

## Consent for publishing

All the authors were agreed to the publication of the present research work in this journal.

## Author’s contribution

Dr. Saima Mehar and Ms. Iqra Anum had performed all data collection and study design that was incorporated in this manuscript. While manuscript writing, laboratory work and all statistical analysis was performed by Dr. Zubia Masood and Dr. Sofia Alvi, Dr. Wali Khan, Muhhamad Kabir, Tauseef Ahmed and Naseem Rafiq. Analysis of heavy metals in fish samples on Atomic absorption spectroscopy (AAS) was performed in collaboration with Dr. Fawad Ahmad.

## Declaration of Competing Interest

The authors declare that they have no known competing financial interests or personal relationships that could have appeared to influence the work reported in this paper.

## References

[b0005] Abass O., Muhammad A., Mada S., Mohammed A., Abdulahi H., Mahmoud K. (2012). Nutrient composition of *Tilapia zilli, Hemisynodontis membranacea, Clupea harengus* and *Scomber scombrus* consumed in zaria. World J. Life Sci. Med. Res..

[b0010] Abdel-Khalek A.A., Badran S.R., Marie M.A.S. (2020). The efficient role of rice husk in reducing the toxicity of iron and aluminum oxides nanoparticles in Oreochromis niloticus: hematological, bioaccumulation, and histological endpoints. Water Air Soil Pollut..

[b0015] Ademoroti C.M.O. (1996). Standard methods for water and effluents analysis.

[b0020] Adewumi A., Adewole H.A., Olaleye V.F. (2014). Proximate and elemental composition of the fillets of some fish species in Osinmo Reservoir, Nigeria. Agric. Biol. J. N. Am..

[b0025] Ahmed Q., Ali Q.M., Bat L. (2017). Assessment of heavy metals concentration in Holothurians, sediments and water samples from coastal areas of Pakistan (northern Arabian Sea). J. Coast. Life Med..

[b0030] Al-Busaidi M., Yesudhason P., Al-Mughairi S., Al-Rahbi W.A.K., Al-Harthy K.S., Al-Mazrooei N.A., AlHabsi S.H. (2011). Toxic metals in commercial marine fish in Oman with reference to national and international standard. Chemosphere.

[b0035] Ali H., Khan E., Ilahi I. (2019). Environmental chemistry and ecotoxicology of hazardous heavy metals: environmental persistence, toxicity, and bioaccumulation. J. Chem..

[b0040] Al-Kahtani A.M. (2009). Accumulation of heavy metal in Tilapia fish (Orechromis niloticus) from Al-Khadod spring, Al-Hassa, Saudi Arabia. Am. J. Appl. Sci..

[b0045] American Public Health Association (APHA), 2005. Standard methods for the examination of water and waste analysis (21st ed.). American water works association/water environment Federation, Washington D.C, pp. 289.

[b0050] Ayanda I.O., Ekhator U.I., Bello O.A. (2019). Determination of selected heavy metal and analysis of proximate composition in some fish species from Ogun River, Southwestern Nigeria. Heliyon.

[b0055] Ayas Z., Ekmekci G., Yerli S.V., Ozmen M. (2007). Heavy metal accumulation in water, sediments and fishes of Nallihan Bird Paradise, Turkey. J. Environ. Biol..

[b0060] Aycicek M., Kaplan O., Yaman M. (2008). Effect of cadmium on germination, seedling growth and metal contents of sunflower (Helianthus annus L.). Asian J. Chem..

[b0065] Carla, S., Imar, M., Carlos, R., 2004. Metals in sport fish tissues of Jobos bay a National Estuarine Research Reserve in Pucrto Rico. The Society of Environmental Toxicology and Chemistry 25th Annual Meeting in North America.

[b0070] Da Silva F.J.G., Gouveia R.M. (2020). Cleaner Production.

[b0075] FAO/WHO, 1989. National Research Council Recommended Dietary Allowances (10th ed.). National Academy Press. Washington, DC., USA.

[b0080] Fatima S., Muzamma I.M., Rehman A., Rustam S.A., Shehzadi Z., Mehmood A., Waqar M. (2020). Water pollution on heavy metals and its effects on fishes. Int. J. Fish. Aquat. Stud..

[b0085] Flores, M., Roselle-Tuzon, M., Bedia, A.C.E., Fortuna, J.L. 2017. Utilization of Marine Fishes (Indian Mackerel (Rastrelliger kanagurta), Mackerel Scad (Decaoterus macarellus), and Surgeonfish (Acanthuridae)) for Fish Longganisa. DLSU Research Congress 2017.

[b0090] Food and Agriculture Organization (FAO), 1983. Compilation of Legal Limits for Hazardous Substances in Fish and Fishery Products. FAO Fishery Circular No (1983), pp. 464.

[b0095] Fu Z., Xi S. (2020). The effects of heavy metals on human metabolism. Toxicol. Mech. Methods.

[b0100] Griboff J., Horacek M., Wunderlin D., Monferran M. (2018). Bioaccumulation and trophic transfer of metals, As and Se through a freshwater food web affected by antrophic pollution in Córdoba, Argentina. Ecotoxicol. Environ. Saf..

[b0105] Imar M., Carlos R. (2011). Metal in sport fish tissue of jobos Bay. National Estuarine Research Reserve Pucrto Rico.

[b0110] Isangedighi I.A., David G.S. (2019). Heavy metals contamination in fish: effects on human health. J. Aquat. Sci. Mar. Boil..

[b0115] Islam M.S., Ahmed M.K., Habibullah-Al-Mamun M. (2015). Determination of heavy metals in fish and vegetables in Bangladesh and health implications. Hum. Ecol. Risk Assess. An. Int. J..

[b0120] Karadede H., Oymak S.A., Ünlü E. (2004). Heavy metals in mullet, Liza abu, and catfish, Silurus triostegus, from the Atatürk Dam Lake (Euphrates). Turkey. Environ. Int..

[b0125] Kaur S., Khera S.K., Kondal J.K. (2018). Effect of water contaminated with heavy metals on histopathology of freshwater catfish, *Clarias batrachus*. Int. J. Chem. Stud..

[b0130] Khalaf M.A., Al-Najjar T., Alawi M., Disi A.A. (2012). Levels of trace metals in three fish species Decapterus macrellus, Decapterus macrosoms and Decapterus russelli of the family carangidae from the Gulf of Aqaba, Red Sea, Jordan. Nat. Sci..

[b0135] Khalek A.A.A., Elhaddad E., Mamdouh S., Marie M.A.S. (2016). Assessment of Metal Pollution around Sabal Drainage in River Nile and its Impacts on Bioaccumulation Level, Metals Correlation and Human Risk Hazard using Oreochromis niloticus as a Bioindicator. Turkish J. Fish. Aquat. Sci..

[b0140] Kumar M., Gupta N., Ratn A., Awasthi Y., Prasad R., Trivedi A., Trivedi S.P. (2020). Biomonitoring of heavy metals in river ganga water, sediments, plant, and fishes of different trophic levels. Biol. Trace Elem. Res..

[b0145] Moiseenko T.I., Morgunov B.A., Gashkina N.A., Megorskiy V.V., Pesiakova A.A. (2018). Ecosystem and human health assessment in relation to aquatic environment pollution by heavy metals: case study of the Murmansk region, northwest of the Kola Peninsula, Russia. Environ. Res. Lett..

[b0150] Moiseenko T.I., Gashkina N.A. (2020). Distribution and bioaccumulation of heavy metals (Hg, Cd and Pb) in fish: Influence of the aquatic environment and climate. Environ. Res. Lett..

[b0155] Monteiro D.A., Thomaz J.M., Rantin F.T., Kalinin A.L. (2013). Cardiorespiratory responses to graded hypoxia in the neotropical fish matrinxã (Brycon amazonicus) and traíra (Hoplias malabaricus) after waterborne or trophic exposure to inorganic mercury. Aquat. Toxicol..

[b0160] Nachman K.E., Raber G., Francesconi K.A., Navas-Acien A., Love D.C. (2012). Arsenic species in poultry feather meal. Sci. Total Environ..

[b0165] Nagajyoti P.C., Lee K.D., Sreekanth T.V.M. (2010). Heavy metals, occurrence and toxicity for plants: a review. Environ. Chem. Lett..

[b0170] Offor I.F., Ehir R.C., Njoku C.N. (2014). Proximate nutritional analysis and heavy metal composition of Dried Moringa Oleifera leaves from Oshiri Onicha L.G.A., Ebonyi State, Nigeria. IOSR J. Environ. Sci. Toxicol. Food Technol..

[b0175] Olusola J.O., Aiyesanmi A.F. (2005). Levels of heavy metal in some selected fish species inhabiting ondo state coastal waters, Nigeria. J. Environ. Anal. Toxicol..

[b0180] Pal D., Maiti S.K. (2018). Seasonal variation of heavy metals in water, sediment, and highly consumed cultured fish (*Labeo rohita* and *Labeo bata*) and potential health risk assessment in aquaculture pond of the coal city, Dhanbad (India). Environ. Sci. Pollut. Res..

[b0185] Raatz S.K., Silverstein J.T., Jahns L., Picklo M.J. (2013). Issues of fish consumption for cardiovascular disease risk reduction. Nutrients.

[b0190] Radulescu C., Dulama I.D., Stihi C., Ionita I., Chilian A., Necula C., Chelarescu E.D. (2014). Determination of heavy metal levels in water and therapeutic mud by atomic absorption spectrometry. Rom. J. Phys..

[b0195] Rahman M.S., Molla A.H., Saha N., Rahman A. (2012). Study on heavy metals levels and its risk assessment in some edible fishes from Bangshi River, Savar, Dhaka, Bangladesh. Food Chem..

[b0200] Rainbow P.S. (2018). Heavy Metals in the Marine Environment.

[b0205] Sarkar B., Islam A. (2020). Drivers of water pollution and evaluating its ecological stress with special reference to macrovertebrates (fish community structure): a case of Churni River, India. Environ. Monit. Assess..

[b0210] Siddique A., Mumtaz M., Zaigham N.A., Mallick K.A., Saied S., Zahir E., Khwaja H.A. (2009). Heavy metal toxicity levels in the coastal sediments of the Arabian Sea along the urban Karachi (Pakistan) region. Mar. Pollut. Bull..

[b0215] Tashla T., Žuža M., Kenjveš T., Prodanović R., Soleša D., Bursić V., Puvača N. (2018). Fish as an important bio-indicator of environmental pollution with persistent organic pollutants and heavy metals. J. Agron. Technol. Eng. Manag..

[b0220] Titcomb M. (1972).

[b0225] Vosylienė M.Z., Jankaitė A. (2006). Effect of heavy metal model mixture on rainbow trout biological parameters. Ekologija.

[b0230] Yeannes M.I., Almandos M.E. (2003). Estimation of fish proximate composition starting from water content. J. Food Compos. Anal..

[b0235] Yousafzai M.A., Sarij M., Ahmed H., Chivers P.D. (2012). Bioaccumulation of heavy metal in common crap: implication for human health. Pak. J. Zool..

[b0240] Yousif R., Choudhary M.I., Ahmed S., Ahmed Q. (2021). Bioaccumulation of heavy metals in fish and other aquatic organisms from Karachi Coast, Pakistan. Nusantara Bioscie..

